# Shifting Perspectives: The Importance of Assessing Suicide Risk in Medically Ill Patients Without Psychiatric History

**DOI:** 10.1192/j.eurpsy.2025.2367

**Published:** 2025-08-26

**Authors:** V. H. Santos, F. M. Tehrani, M. Pires, R. P. Andrade, B. Sousa, Z. C. e Sá, T. Carvalhão, S. Fontes

**Affiliations:** 1 Department of Psychiatry and Mental Health, Cova da Beira Local Health Unit; 2 Faculty of Health Sciences, University of Beira Interior, Covilhã, Portugal; 3 Child and Adolescent Mental Health Center, Mental Health Services in the Capital Region of Denmark, Copenhagen, Denmark; 4 Department of Psychiatry and Mental Health, Guarda Local Health Unit, Guarda; 5 Department of Psychiatry and Mental Health, Viseu Dão-Lafões Local Health Unit, Viseu, Portugal

## Abstract

**Introduction:**

Chronic medical conditions are increasingly recognized as significant contributors to suicide risk, especially in patients without prior psychiatric diagnoses (Østergaard *et al*. JAMA Psychiatry 2024). This case examines the psychiatric impact in a 65-year-old male admitted to general surgery for abdominal pain, who subsequently underwent an ileocolectomy for suspected gastrointestinal malignancy.

**Objectives:**

To explore the psychiatric impact of medical morbidity on suicide risk, emphasizing recent findings suggesting heightened attention for patients without a psychiatric history.

**Methods:**

The patient, with no prior psychiatric history, was observed by liaison psychiatry after verbalizing suicide ideation without intent or plan. The ideation was associated with worsening mood, particularly over the past week. Emotional distress escalated after perceived medical setbacks and was compounded by familial dynamics, particularly the wife’s expressed anxiety. A diagnosis of adjustment disorder with depressive symptoms was considered, with initial treatment involving mirtazapine and psychosocial support.

**Results:**

The case reflects evidence supporting the idea that suicide risk may follow a dose-response pattern based on the disability burden in patients without prior psychiatric history (Østergaard *et al*. JAMA Psychiatry 2024). This suggests clinicians may need to be particularly vigilant in medically ill patients without a psychiatric background, as their suicide risk may increase as disability burdens mount – contrary to the understandable and maybe more intuitive focus on those with established psychiatric diagnoses.

**Conclusions:**

This case highlights the importance of thorough suicide risk evaluation in patients without psychiatric histories, particularly following a major medical diagnosis. While suicide risk remains high in psychiatric patients, clinicians must be equally or even more vigilant with medically ill patients without psychiatric histories. Psychiatric care should be integrated early, with attention to the timing of suicide risk, the disability burden, and psychosocial stressors. This highlights the need for careful monitoring and early intervention, particularly in the acute phase following medical complications, where risk evaluation may be more nuanced.

**Disclosure of Interest:**

None Declared

